# Automated Synthesis and Initial Evaluation of (4′-Amino-5′,8′-difluoro-1′H-spiro[piperidine-4,2′-quinazolin]-1-yl)(4-[^18^F]fluorophenyl)methanone for PET/MR Imaging of Inducible Nitric Oxide Synthase

**DOI:** 10.1155/2021/9996125

**Published:** 2021-07-08

**Authors:** Skye Hsin-Hsien Yeh, Wen-Sheng Huang, Chuang-Hsin Chiu, Chuan-Lin Chen, Hui-Ting Chen, Dae Yoon Chi, Zhengxing Ge, Tsung-Hsun Yu, Pao-Yeh Wang, Yu-Yeh Kuo, Chun-Tse Hung, Geng-Ying Li, Chi-Wei Chang

**Affiliations:** ^1^Brain Research Center, National Yang Ming Chiao Tung University, Taipei, Taiwan; ^2^Department of Nuclear Medicine, Taipei Medical University Hospital, Taipei, Taiwan; ^3^Department of Nuclear Medicine, Taipei Veterans General Hospital, Taipei, Taiwan; ^4^Department of Nuclear Medicine, Tri-Service General Hospital, Taipei, Taiwan; ^5^Department of Biomedical Imaging and Radiological Sciences, National Yang Ming Chiao Tung University, Taipei, Taiwan; ^6^Department of Pharmacy, National Yang Ming Chiao Tung University, Taipei, Taiwan; ^7^Department of Fragrance and Cosmetic Science, Kaohsiung Medical University, Taiwan; ^8^FutureChem Co. Ltd., Seoul, Republic of Korea; ^9^Jiangsu Huayi Technology Co. Ltd., Jiangsu, China; ^10^Department of Biomedical Engineering and Environmental Sciences, College of Nuclear Science, National Tsing Hua University, Hsinchu, Taiwan; ^11^Department of Medical Imaging and Radiological Sciences, Institute of Radiological Sciences, Tzu Chi University of Science and Technology, Hualien, Taiwan

## Abstract

**Background:**

Inducible nitric oxide synthase (iNOS) plays a crucial role in neuroinflammation, especially microglial activity, and may potentially represent a useful biomarker of neuroinflammation. In this study, we carefully defined a strategic plan to develop iNOS-targeted molecular PET imaging using (4′-amino-5′,8′-difluoro-1′H-spiro[piperidine-4,2′-quinazolin]-1-yl)(4-fluorophenyl)methanone ([^18^F]FBAT) as a tracer in a mouse model of lipopolysaccharide- (LPS-) induced brain inflammation.

**Methods:**

An *in vitro* model, murine microglial BV2 cell line, was used to assess the uptake of [^18^F]FBAT in response to iNOS induction at the cellular level. *In vivo* whole-body dynamic PET/MR imaging was acquired in LPS-treated (5 mg/kg) and control mice. Standard uptake value (SUV), total volume of distribution (*V*_t_), and area under the curve (AUC) based on the [^18^F]FBAT PET signals were determined. The expression of iNOS was confirmed by immunohistochemistry (IHC) of brain tissues.

**Results:**

At the end of synthesis, the yield of [^18^F]FBAT was 2.2–3.1% (EOS), radiochemical purity was >99%, and molar radioactivity was 125–137 GBq/*μ*mol. *In vitro*, [^18^F]FBAT rapidly and progressively accumulated in murine microglial BV2 cells exposed to LPS; however, [^18^F]FBAT accumulation was inhibited by aminoguanidine, a selective iNOS inhibitor. *In vivo* biodistribution studies of [^18^F]FBAT showed a significant increase in the liver and kidney on LPS-treated mice. At 3 h postinjection of LPS, *in vivo*, the [^18^F]FBAT accumulation ratios at 30 min post intravenous (i.v.) radiotracer injection for the whole brain, cortex, cerebellum, and brainstem were 2.16 ± 0.18, 1.53 ± 0.25, 1.41 ± 0.21, and 1.90 ± 0.12, respectively, compared to those of mice not injected with LPS. The mean area under the curve (AUC_0-30min_), total volume of distribution (*V*_t_, mL/cm^3^), and *K*_i_ (influx rate) of [^18^F]FBAT were 1.9 ± 0.21- and 1.4 ± 0.22-fold higher in the 3 h LPS group, respectively, than in the control group. In the pharmacokinetic two-compartment model, the whole brain *K*_i_ of [^18^F]FBAT was significantly higher in mice injected with LPS compared to the control group. Aminoguanidine, selective iNOS inhibitor, pretreatment significantly reduced the AUC_0-30min_ and *V*_t_ values in LPS-induced mice. Quantitative analysis of immunohistochemically stained brain sections confirmed iNOS was preferentially upregulated in the cerebellum and cortex of mice injected with LPS.

**Conclusion:**

An automated robotic method was established for radiosynthesis of [^18^F]FBAT, and the preliminary *in vitro* and *in vivo* results demonstrated the feasibility of detecting iNOS activity/expression in LPS-treated neuroinflammation by noninvasive imaging with [^18^F]FBAT PET/MRI.

## 1. Introduction

Nitric oxide (NO) is a critical, unique mediator of a variety of physiological and pathological processes [1, 2]. Two constitutively expressed nitric oxide synthase (NOS) isozymes—neuronal NOS (nNOS) and endothelial NOS (eNOS)—and the inducible isozyme iNOS generate NO by oxidizing L-arginine to L-citrulline [3, 4]. NOS are detected in a variety of tissues and participate in distinct physiological functions: NO produced by nNOS, which is mainly expressed in the peripheral nerves and brain, acts as a neuromodulator and neurotransmitter, whereas eNOS is primarily expressed in vascular endothelial cells and plays a role in regulation of blood pressure [1, 5]. In contrast, iNOS is not constitutively expressed in cells and is only induced when cells are stimulated, typically by proinflammatory cytokines and/or bacterial lipopolysaccharide (LPS) [6, 7]. In the central nervous system (CNS), iNOS is expressed in a variety of glial cells, such as astrocytes and microglia. Microglia are considered the “macrophages of the brain.” Induction of iNOS expression is thought to be a specific marker of the M1 macrophage and microglial phenotypes [8] and mediates host defense and inflammatory processes induced by various stimuli. The levels of iNOS expression are limited in neurons compared to glial cells.

Nitric oxide (NO), the end product of metabolism catalyzed by the iNOS, is an important mediator of a variety of inflammatory diseases. When induced, iNOS generates significant amounts of NO (in the micromolar range), until the enzyme is degraded, sometimes hours later [9]. Production of NO by iNOS helps to defend against invading pathogens and thus plays critical roles in the inflammatory response and innate immune system. However, production of inappropriately high NO concentrations due to overexpression or dysregulation of iNOS can result in toxic effects and is associated with a variety of human diseases, including septic shock, cardiac dysfunction, pain, diabetes, and cancer [6].

LPS is frequently employed to induce neuroinflammation in animal models. LPS induces the release of immunologically active mediators implicated in inflammatory diseases—including TNF-*α*, IL-6, IL-8, and IL-1*β*—from host cells, especially mononuclear phagocytes [10]. Moreover, NO itself can also mediate inflammatory processes, as inhibiting iNOS activity and NO generation reduced the progression and severity of inflammatory disease in experimental models. Therefore, development of a radiolabeled iNOS radiopharmaceutical to noninvasively assess iNOS protein concentrations in living tissues—as a specific biomarker of iNOS activity—would advance our knowledge of NO-related diseases and may help to identify novel treatments.

Several PET imaging tracers based on iNOS inhibitors have been developed for noninvasive assessment of iNOS levels in living tissues, including N-omega-nitro-L-arginine [^11^C]methyl ester ([^11^C]L-NAME) (iNOS IC_50_ = 20 *μ*M) [11], S-[^11^C]methylisothiourea ([^11^C]MITU, iNOS IC_50_ = 3.0 *μ*M, 2.3-fold selectivity vs. eNOS), and S-(2-[^18^F]fluoroethyl)isothiourea ([^18^F]FEITU, iNOS IC_50_ = 0.14 *μ*M, 9-fold selectivity vs. eNOS) [12, 13]. Furthermore, 8-fluoro-3-(4-fluorophenyl)-3,4-dihydro-1-isoquino-linamine (FFDI) has a high affinity (IC_50_ = 0.16 *μ*M) and selectivity for iNOS (100-fold selectivity for iNOS vs. nNOS, ~1000-fold vs. eNOS) [14]. 8-Fluoro-3-(4-[^18^F]fluorophenyl)-3,4-dihydro-1-isoquinolinamine ([^18^F]FFDI), which is based on FFDI, has been reported to act as a PET radiotracer for iNOS [15]. Moreover, [^18^F]6-(2-fluoropropyl)-4-methyl-pyridin-2-amine ([^18^F]iNOS-9) was used as a PET tracer to image the activation of iNOS in a mouse model of lung inflammation [16] (among structures described above are shown in Suppl. Fig. 1).

Because FFDI demonstrated similar or even superior potency in the cells to that of the standard iNOS inhibitors such as L-NMMA, FEITU, or MITU [14], therefore, in this report, based on FFDI, we developed (4′-amino-5′,8′-difluoro-1′H-spiro[piperidine-4,2′-quinazolin]-1-yl)(4-[^18^F]fluorophenyl)methanone ([^18^F]FBAT) as a tracer to detect iNOS expression in mice injected with lipopolysaccharide (LPS) to induce brain inflammation. We assessed the potential of [^18^F]FBAT as a prognostic marker for the progression of neuroinflammatory disorders. [^18^F]FBAT was radiosynthesized from aryl pinacol boronates via the copper-mediated aromatic nucleophilic radiofluorination at an acceptable radiochemical yield and molar activity. Moreover, the [^18^F]FBAT PET findings were correlated with the results of immunohistochemistry. We anticipate that this study will not only advance our understanding of neuroinflammation diseases from the perspective of molecular imaging but also potentially help to develop novel tools to diagnose and guide the management of these diseases during the early progressive stages in humans.

## 2. Materials and Methods

### 2.1. Reagents and Equipment

A Scanditronix MC 17 cyclotron was used for radionuclide production. The radiosynthesis was performed using a robotic system (ANATEC, Uppsala, Sweden). Radiochemical yields were determined with a dose calibrator (Capintec CRC-712M, Florham Park, NJ, USA). The radioactive mixtures were purified via semipreparative high-performance liquid chromatography (HPLC) using a Varian 230 pump, Varian 325 UV/VIS detector (Varian Corp., Palo Alto, CA, USA), a pin-diode radiodetector (Bioscan 2000; Capintec Inc., Florham Park, NJ, USA), and a Rotavapor (Büchi, Flawil, Switzerland). NMR spectra of [^19^F]FBAT reference standard were determined by NMR spectrophotometer (Bruker, Bruker 300 Ultrashield, USA). MS of [^19^F]FBAT reference standard were determined by LC/MS (Agilent Technologies, 6120 Quadrupole, USA). NMR spectra of [^18^F]FBAT precursor were determined by NMR spectrophotometer (Varian Inc./California, 400 MR, 400 MHz, USA). ESI-Mass of [^18^F]FBAT precursor were determined by LC/MS (Varian Inc./California, Varian 500-MS LC Ion Trap, USA). ESI-Mass of [^18^F]FBAT precursor were determined by LC/MS (Varian Inc./California, Varian 500-MS LC Ion Trap, USA). Quality control of the final product was conducted by analytical HPLC using a Varian 230 pump, Varian 325 UV/VIS (254 nm) detector (Varian Corp.), and Packard Radiomatic 150TR (BT cell) radiodetector (PerkinElmer Inc., CT, USA). The amount of organic solvent was determined by gas chromatography (Varian Corp.).

Unless otherwise stated, all chemicals were obtained from Sigma-Aldrich Chemical Co. (St. Louis, MO, USA) or Merck Co. (Darmstadt, Germany) and used without further purification. Silica plus Sep-Pak cartridges were obtained from Waters Corporation (Milford, MA, USA).

**Scheme 1:**
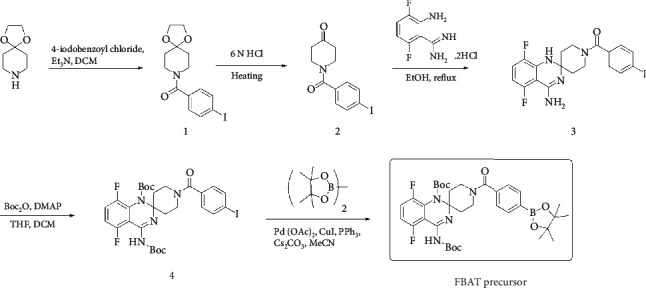
Synthetic route to precursor.

**Scheme 2:**
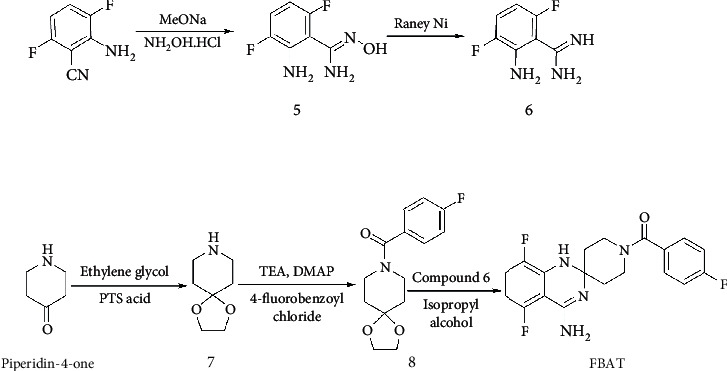
Synthetic route to FBAT standard.

The synthetic routes of the precursor and authentic FBAT for [^18^F]FBAT are presented in Schemes 1 and 2.

### 2.2. Chemistry

#### 2.2.1. Synthesis of (4-Iodophenyl)(1,4-dioxa-8-azaspiro[4.5]decan-8 yl)methanone (1)

Boc_2_O (1.1 g, 5.03 mmol) dissolved in tetrahydrofuran (10 mL) was added to a solution of 1,4-dioxa-8-azaspiro[4.5]decane (2 g, 14.0 mmol) and dichloromethane (130 mL, anhydrous) under argon gas in an ice-bath; then after 10 min, a solution of 4-iodobenzoyl chloride (3.72 g, 14.0 mmol) in dichloromethane (10 mL) was added, and the reaction mixture was stirred for 30 min at room temperature. Saturated NaHCO_3_ solution was added, and the organic layer was separated and dried over anhydrous Na_2_SO_4_ and concentrated. The residue was purified by silica-gel column chromatography (60% ethyl acetate/*n*-hexane) to yield 5 g (96%) of *compound 1* as a white solid. ^1^H NMR (400 MHz, CDCl_3_) *δ* = 7.75 (d, *J* = 8.0 Hz, 2H), 7.13 (d, *J* = 8.0 Hz, 2H), 3.98 (s, 4H), 3.82 (br s, 2H), 3.46 (br s, 2H), 1.78 (br s, 2H), 1.63 (br s, 2H); MS (ESI+) *m/z* (%) 374.1 [M+H]^+^, 399.2 [M+Na]^+^.

#### 2.2.2. Synthesis of 1-(4-Iodobenzoyl)piperidin-4-one (2)

A solution of *compound 1* (3 g, 8.0 mmol) in hydrochloric acid (50 mL, 6 N (aq.)) was heated for 2 h at 90°C, cooled to room temperature, adjusted pH 10~11 with sodium hydroxide solution (10 *wt%*), diluted with dichloromethane and the organic layer was separated, washed with brine, dried over anhydrous Na_2_SO_4_, and concentrated. The residue was purified by silica-gel column chromatography (50% ethyl acetate/*n*-hexane) to yield 1.3 g (49%) of *compound 2* as a white solid. ^1^H NMR (400 MHz, CDCl_3_) *δ* = 7.80 (d, *J* = 8.8 Hz, 2H), 7.22 (d, *J* = 8.4 Hz, 2H), 3.86 (br s, 4H), 2.50 (br s, 4H); MS (ESI+) *m/z* (%) 330.1 [M+H]^+^, 352.0 [M+Na]^+^.

#### 2.2.3. Synthesis of Compound 3

A solution of *compound 1* (404 mg, 1.23 mmol) and 2-amino-3,6-difluorobenzamidine dihydrochloride (300 mg, 1.23 mmol) in ethanol (5 mL) was refluxed overnight and cooled to room temperature, and the solid precipitate was filtered and purified by silica-gel column chromatography (5~10% methanol/dichloromethane) to yield 450 mg (76%) of *compound 3* as a yellow solid. ^1^H NMR (400 MHz, CDCl_3_) *δ* = 7.74 (d, *J* = 8.0 Hz, 2H), 7.14 (d, *J* = 8.0 Hz, 2H), 7.00 (td, *J* = 4.8 Hz, 9.2 Hz, 1H), 6.33 (m, 1H), 4.22 (br s, 1H), 3.70-3.45 (m, 3H), 2.20-1.90 (m, 2H), 1.70-1.60 (m, 2H); MS (ESI+) *m/z* (%) 483.1 [M+H]^+^.

#### 2.2.4. Synthesis of Compound 4

Boc_2_O (1.1 g, 5.03 mmol) dissolved in tetrahydrofuran (10 mL) was added to a solution of *compound 3* (485 mg, 1.01 mmol) and DMAP (123 mg, 1.01 mmol) in dichloromethane (10 mL, anhydrous) in an ice bath. The reaction mixture was stirred overnight at room temperature, the solvent was evaporated under reduced pressure, and the residual solution was purified by silica-gel column chromatography (40% ethyl acetate/*n*-hexane) to yield 432 mg (70%) of *compound 4* as a foamy yellow solid. ^1^H NMR (400 MHz, CDCl_3_) *δ* = 7.76 (d, *J* = 6.8 Hz, 2H), 7.16 (d, *J* = 6.4 Hz, 2H), 6.99 (td, *J* = 4.4 Hz, 9.2 Hz, 1H), 6.30 (m, 1H), 4.39 (br s, 1H), 4.16 (s, 1H), 3.70-3.52 (m, 2H), 3.50-3.36 (m, 1H), 2.38-2.26 (b, 1H), 2.18-2.08 (m, 1H), 1.80-1.56 (m, 2H), 1.40 (s, 18H); MS (ESI-) *m/z* (%) 681.7 [M-H]^+^.

#### 2.2.5. Synthesis of [^18^F]FBAT Precursor (Boron N-2-Protected Precursor) (*tert*-Butyl-4′-((*tert*-butoxycarbonyl)amino)-5′,8′-difluoro-1-(4-(4,4,5,5-tetramethyl-1,3,2-dioxaborolan-2-yl)benzoyl)-1′H-spiro[piperidine-4,2′-quinazoline]-1′-carboxylate)

A solution of *compound 4* (360 mg, 0.53 mmol), palladium acetate (2.4 mg, 11 *μ*mol), copper iodide (20 mg, 0.11 mmol), triphenylphosphine (2.8 mg, 11 *μ*mol), cesium carbonate (258 mg, 0.79 mmol), and bis(pinacol)diboron (200 mg, 0.79 mmol) in acetonitrile (5 mL) was stirred for 16 h at room temperature, filtered through a celite pad, and concentrated under reduced pressure. The residue was diluted with dichloromethane and water; the organic layer was separated, washed with brine, dried over anhydrous Na_2_SO_4_, and concentrated. The residue was purified by silica-gel column chromatography (50% ethyl acetate/*n*-hexane) to yield 176 mg (49%) of boron N-2-protected precursor as a light-yellow solid. ^1^H NMR (400 MHz, CDCl_3_) *δ* = 7.85 (d, *J* = 7.6 Hz, 2H), 7.41 (d, *J* = 7.6 Hz, 2H), 6.99 (td, *J* = 4.8 Hz, 10.0 Hz, 1H), 6.29 (td, *J* = 3.6, 10.0 Hz, 1H), 4.40 (br s, 1H), 4.16 (s, 1H), 3.68-3.40 (m, 3H), 2.38-2.26 (m, 1H), 2.16-2.06 (m, 1H), 1.80-1.60 (m, 2H), 1.42 (s, 9H), 1.38 (s, 9H), 1.35 (s, 12H); MS (ESI-) *m/z* (%) 681.4 [M-H]^+^ (Suppl. Fig. 2A-B).

#### 2.2.6. Synthesis of Compound 5

The solution of 20 mL of methanol and metal sodium (0.364 g, 15.8 mmol) was placed into a 250 mL three-port bottle under N_2_ atmosphere to prepare sodium methoxide, and hydroxylamine hydrochloride (1.100 g, 15.8 mmol) was added, the mixture was stirred, 2-amino-3,6-difluorobenzonitrile (2.440 g, 15.8 mmol) was added gradually, and then, the solution was allowed to equilibrate to room temperature and refluxed for 18 h.

The mixture was concentrated to oil that partitioned between ethyl acetate and 10% sodium hydroxide solution. The basic phase was separated and extracted three times with ethyl acetate, and the organic solution was washed three times with saturated brine and dried over sodium sulfate. The solvent was evaporated, and the product was purified by flash column chromatography on silica gel and eluted in DCM/MeOH = 100/1‐50/1 to obtain *compound 5* as a white solid product (1.831 g), yield 61.8%. ^1^H-NMR (CDCl_3_) 6.96-6.88 (1H, m), 6.82 (1H, m), 6.38-6.30 (1H, m), 5.15 (4H, s). LC-MS: calculated for C_7_H_7_F_2_N_3_O, 187.15; found [M+H] 188.1.

#### 2.2.7. Synthesis of Compound 6

*Compound 5* (1.830 g, 9.8 mmol) and wet Raney nickel (ca. 1.002 g) in ethanol (80 mL) were placed in a pressure bottle and stirred under 0.3 MPa hydrogen at 60°C for 8 h. The catalyst was removed by filtration and the solvent evaporated to give the product as oil, which was dissolved in ethanol (5 mL). HCl (1 N) in ether was added under stirring, and solid *compound 5* was collected by filtration to give an off-white powder (2.028 g), yield almost 100%. ^1^H-NMR (CDCl_3_) 9.48 (2H, s), 9.24 (2H, s), 7.26-7.18 (1H, m), 6.48-6.41 (1H, m). LC-MS: calculated for C_7_H_7_F_2_N_3_, 171.15; found [M+H] 172.0.

#### 2.2.8. Synthesis of Compound 7

A mixture of 4-piperidone monohydrate hydrochloride (30 g, 0.195 mol), ethylene glycol (13 g, 0.209 mol), and *p*-toluenesulfonic acid (1.85 g, 0.011 mol) was heated and refluxed in 350 mL of toluene for 6 h; water from the reaction was removed by azeotropic distillation of the water separator. After cooling to room temperature, 2 M aqueous sodium hydrate was added to adjust the pH to 14, the organic layer was separated, the aqueous layer was extracted with toluene, and the toluene layer was combined, followed by drying on anhydrous sodium carbonate. After removing sodium carbonate by filtration, the solvent was distilled off under reduced pressure, and the residue was distilled under reduced pressure to obtain a colorless liquid (25.1 g; yield, 79.2%). ^1^H-NMR (CDCl_3_) 3.96 (4H, s), 2.94-2.90 (4H, t), 1.68-1.64 (4H, t), 1.42 (1H, s). LC-MS: calculated for C_7_H_13_NO_2_, 143.19; found [M+H] 144.1.

#### 2.2.9. Synthesis of Compound 8

*Compound 7* (1.001 g, 6.98 mmol), triethylamine (1.555 g, 15.37 mmol), DMAP (0.085 g, 0.69 mmol), and methylene chloride (20 mL) were added to a three-mouth bottle in an ice-salt bath under N_2_ atmosphere. A methylene chloride solution of 4-fluorobenzoyl chloride (1.426 g, 7.68 mmol) was added dropwise while keeping the temperature below 0°C; then, the solution was equilibrated to room temperature and stirred overnight. The DCM was diluted and washed with brine, and the organic extract was dried over magnesium sulfate and filtered. The solvent was evaporated under reduced pressure, and the remaining mixture was separated, dried, and concentrated. The crude product was purified by flash column chromatography on silica gel and eluted with DCM/MeOH = 100/1 to obtain *compound 8* as an off-white solid (1.610 g). The product was recrystallized in ethyl acetate and *n*-hexane to obtain 1.152 g of white crystals (yield, 62.1%). ^1^H-NMR (CDCl_3_) 7.41-7.39 (2H, m), 7.12-7.09 (2H, t), 3.99 (4H, s), 3.82 (2H, s), 3.51 (2H, s), 1.72-1.69 (4H, m). LC-MS: calculated for C_14_H_16_FNO_3_, 265.28; found [M+H] 266.0.

#### 2.2.10. Synthesis of FBAT Standard ([^19^F]FBAT)

*Compound 6* hydrochloride (0.200 g, 0.963 mmol), *compound 8* (0.281 g, 1.059 mmol), and 20 mL isopropyl alcohol were added to a 50 mL single-neck bottle under N_2_ atmosphere. The reaction mixture was refluxed overnight and cooled to precipitate a large amount of yellow solid. The mixture was filtered, and the filter cake was washed with ether to obtain 0.382 g of yellow solid. The solid was dissolved in 10 mL of methanol and adjusted to pH 8 with ammonia, and the mixture was extracted twice with dichloromethane. The organic phase was washed with saturated sodium chloride solution, dried over magnesium sulfate, filtered, and concentrated under reduced pressure. The crude product was purified by flash column chromatography on silica gel, eluted with DCM/MeOH (20/1 *v*/*v*) to obtain the product, which was recrystallized in dichloromethane and *n*-hexane to obtain the product (0.310 g) as an off-white solid (yield, 85.9%). ^1^H-NMR (DMSO) 8.31-8.28 (2H, d), 7.66-7.63 (2H, d), 7.18-7.10 (1H, m), 6.39-6.31 (1H, m), 4.26-4.22 (1H, d), 3.45-3.39 (1H, m), 3.32-3.26 (2H, m), 1.87-1.68 (4H, m). LC-MS: calculated for C_19_H_17_F_3_N_4_O, 374.37; found [M+H] 375.2 (Suppl. Fig. 3A-B).

### 2.3. Radiochemistry

#### 2.3.1. Production of Reactive [^18^F]HF

No-carrier-added (NCA) aqueous [^18^F]F-fluoride was produced with a Scanditronix MC17F cyclotron by proton irradiation of 98% enriched [^18^O]water with 37 *μ*Ah beam current integration. Aqueous [^18^F]HF was delivered with helium, passed through a preconditioned QMA cartridge, and eluted with 550 *μ*L of water eluent containing 50 *μ*g of K_2_CO_3_ and 5 mg of KOTf into a 5 mL Reacti-vial. The water was evaporated using a stream of nitrogen (200 mL/min) at 120°C and coevaporated to dryness with CH_3_CN (7 × 0.5 mL). Cu(II)(OTf)_2_ (7.3 mg) and 0.1 mL pyridine/0.6 mL dry DMF were added to the dried [^18^F]KF, and dry air was delivered to the vial for 1 min.

#### 2.3.2. Production of [^18^F]FBAT

**Figure 1:**
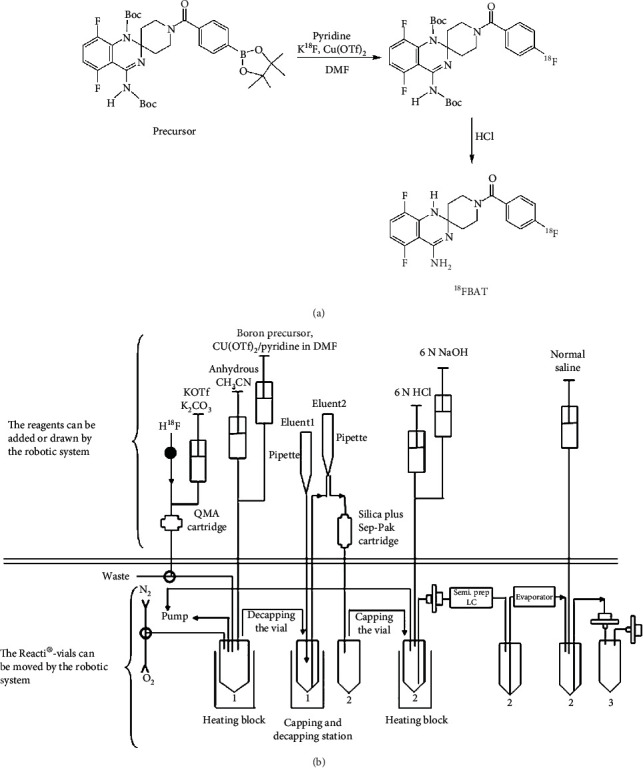
(a) Radiosynthesis scheme of (4′-amino-5′,8′-difluoro-1′H-spiro[piperidine-4,2′-quinazolin]-1-yl)(4-[^18^F]fluorophenyl)methanone ([^18^F]FBAT). (b) Diagram of the automated robotic system for the radiosynthesis of [^18^F]FBAT. Solid circle: one-way magnetic valve; open circle: two-way magnetic valve. Eluent 1: CH_2_Cl_2_; eluent 2: CH_2_Cl_2_/CH_3_OH(3/2); vial 1: labeling vial; vial 2: hydrolysis vial; vial 3: product vial.

The precursor of [^18^F]FBAT (2.0 mg in 0.5 mL of DMF) was added to the dried [^18^F]KF, and dry air was delivered to the vial for 1 min. The labeling reaction was carried out in a heating block station at 110°C for 10 min. The reaction mixture was passed through a silica plus Sep-Pak cartridge that had been preconditioned with 5 mL of CH_2_Cl_2_ to keep the cartridge wet. Then, the vial was rinsed with 1.5 mL of CH_3_OH/CH_2_Cl_2_ (2/3 *v*/*v*), the solution was passed through a silica plus Sep-Pak cartridge, another 1.5 mL of CH_3_OH/CH_2_Cl_2_ was passed through the silica plus Sep-Pak cartridge, and the eluate was reduced to 0.4 mL in a heating block station at 65°C under a stream of nitrogen (200 mL/min) in a vacuum. One milliliter of 6 N HCl was added, the solution was heated at 110°C for 10 min and cooled to room temperature, and 1 mL of 6 N NaOH was added to neutralize the solution. The resulting solution was passed through a 0.22 *μ*m PVDF membrane filter (Millipore) into a 5 mL Reacti-vial. The reaction scheme is shown in Figure 1(a).

#### 2.3.3. Robotic System

All procedures described above were performed using a robotic system (Scanditronix Anatech RB III, Uppsala, Sweden). A diagram of the synthetic system is shown in Figure 1(b). The robotic system included a hand station that gripped the Reacti-vials, a syringe station that dispensed the solutions, a vortex station, a heating block station, cap stations that capped and uncapped the Reacti-vials, a solid phase extraction station for primary column purifications, a movable Reacti-vial rack station that collected the eluted fractions, a filtration station that filtered the raw [^18^F]FBAT product, a filter check station that checked the integrity of the 0.22 *μ*m membrane filters, a solvent rack station, and a water cooling station.

#### 2.3.4. Purification and Formulation of [^18^F]FBAT

Crude [^18^F]FBAT was purified via semipreparative HPLC on a C18 guard column (YMC-Triart, RP-C18, 5 *μ*m, 10 mm × 20 mm) followed by a RP-C18 semipreparative column (YMC-Triart, C18 reverse phase, 5 *μ*m, 120 A°, 250 mm × 20 mm) and eluted with acetonitrile/(0.1% triethylamine/H_2_O) (3/2, *v*/*v*) pH 9.0 at a flow rate of 10 mL/min. The [^18^F]FBAT fraction was transferred to a round-bottomed flask and evaporated to dryness using a rota-evaporator. The formulation was prepared by adding 2 mL of physiological saline (0.9%) to reconstitute the final [^18^F]FBAT product. The saline solution of [^18^F]FBAT was passed through a 0.22 *μ*m filter (Millex-GV, Millipore Corp., Burlington, MI, USA) into a sterile, pyrogen-free vial.

### 2.4. Quality Assurance

The radiochemical purity and molar activity of [^18^F]FBAT were determined via analytical HPLC on a reverse phase column (YMC-Triart, RP-C18, 250 × 4.6 mm, 120 A°, 5 *μ*m, Japan) and eluted with acetonitrile/(0.025% triethylamine/H_2_O) (2/3 *v*/*v*; pH 9.0) at a flow rate of 1 mL/min and detected using a UV-VIS detector (254 nm) and radio detector. The amount of organic solvent was determined by gas chromatography.

### 2.5. Log *P* Measurements

The partition coefficient of FBAT, determined between 1-octanol and 0.02 M phosphate buffer at pH 7.4, was measured by UV spectrophotometer (SPARK 10 M from Tecan) at wavelength 230 nm [17, 18].

### 2.6. In Vitro Assessment of LPS-Dependent Nitric Oxide (NO) Production

Previously, BV2 cells have been shown to respond to LPS stimulation by increasing the expression of iNOS [19] and released nitric oxide (NO) in a concentration-dependent manner [20]. Thus, we investigated the dose-dependent increase in NO production in LPS-challenged BV2 cells.

Briefly, murine microglial BV2 cells [19] were cultured in high-glucose Dulbecco's modified Eagle's medium (DMEM) supplemented with 100 U/mL penicillin. The cells were cultured in 6-well plates (~50,000 cells/well) in a humidified 5% CO_2_ atmosphere at 37°C until ∼60–70% confluent, washed three times with fresh media, and cultured in complete media containing LPS (0, 50, 100, or 200 ng/mL, Sigma-Aldrich, St. Louis, MO, USA). At the indicated time points, the culture media were harvested.

To assay NO, 100 *μ*L aliquots of culture media were added to 100 *μ*L of Griess reagent (1% sulfanilamide in 5% H_3_PO_4_) in 96-well plates, incubated at room temperature for 10 min; then, 50 *μ*L NED Griess reagent (0.1% N-naphthyl-ethylenediamine dihydrochloride) was added and incubated for 10 min, and the absorbance values were determined at 540 nm using a TECAN Sunrise ELISA Reader. Fresh culture medium was used as a blank. The concentrations of nitrite were determined by comparison with a standard curve of sodium nitrite prepared in cell culture media.

### 2.7. In Vitro [^18^F]FBAT Uptake Assay

BV2 cells were cultured as described above, washed three times with fresh medium, pretreated with a selective iNOS inhibitor, aminoguanidine (AMG), (0, 1, or 0.1 mM) for 1 h, and then treated with cell culture media containing LPS and the inhibitor. At the indicated time points, the cells were washed thrice with fresh cell culture media, then incubated in fresh cell culture media continuing the radiotracer [^18^F]FBAT at 0.37 MBq/mL for 15, 30, or 60 min, harvested by gentle scraping, and pelleted by centrifugation at 1500 g for 2 min. The weight of the cell pellet and 0.1 mL of the radioactive supernatant was determined. A Packard 5500 gamma counter (PerkinElmer, Billerica, MA, USA) was used to quantify radioactivity as cpm/g cells or cpm/mL media, respectively. Cell-to-medium radioactivity concentration ratios were calculated and plotted over time to evaluate the kinetics of accumulation of the radiotracer.

iNOS is the enzyme responsible for NO generation in BV2 cells [20, 21]; therefore, accumulation of [^18^F]FBAT in BV2 cells before or after iNOS inhibitor versus NO production was also assessed.

### 2.8. Animals

All animal handling procedures were approved by Yang-Ming University Institutional Animal Care and Use Committee (IACUC No. 1050910), and animal study was performed according to the Guidelines for Animal Experimentation of National Yang-Ming University. For metabolism study, the ten-week-old male Sprague-Dawley rat (255 ± 12.3 g) or eight-week-old male C57BL/6 mice (22 ± 0.4 g) received food and water *ad libitum* and were housed under controlled room temperature (22 ± 2°C) and humidity (55-65%) under a 12 : 12 h light-dark cycle. The dark cycle lasted from 19:00 to 7:00.

We performed a preliminary study to identify the dose of LPS that induces moderate neuroinflammation in mice. Male C57BL/6 mice from BioLASCO (*n* = 18, weight = 25 ± 0.6 g) were intratracheally administered a bolus containing 5, 10, or 15 mg/kg LPS (Sigma-Aldrich; *n* = 6 mice per group). Behavior, breathing rate, appetite, and mortality rate were recorded over 48 h. The group receiving 10 and 15 mg/kg LPS exhibited mild to moderate clinical and physical symptoms of adult respiratory distress syndrome (ARDS) within 24 h of administration of LPS. All animals exhibited chills; the mortality rates for the 5, 10, and 15 mg/kg LPS groups were 16.6%, 50%, and 83%, respectively. The mortality rates reduced to 0%, 40%, and 50%, respectively, when the animals were kept on heating pads until they recovered; these animals were less mobile and had decreased appetite. Thus, 5 mg/kg was determined as a suitable dose of LPS to induce moderate neuroinflammation in mice.

For the imaging experiments, twelve C57BL/6 mice were injected *i.p.* with 0.9% NaCl, eighteen mice were injected *i.p.* with 0.1 mL of LPS in saline (5 mg/kg), and six mice were injected *i.p.* with aminoguanidine (Sigma-Aldrich) 30 min prior to 5 mg/kg LPS*. In vivo* imaging was performed 3 h or 24 h after injection of LPS; then, the mice were immediately humanely euthanized and the brains were removed for immunostaining.

### 2.9. Assessment of Radiolabeled Metabolites in Blood Plasma

Adult Sprague-Dawley rats (*n* = 3) were anesthetized with 2% isoflurane (in oxygen) and injected with 194.87 MBq ± 22.61 MBq of [^18^F]FBAT in the tail vein. Plasma was extracted from heparinized blood samples (0.25 mL) with 3x volumes of acetonitrile and analyzed with radio-HPLC; mobile phase: 40% MeCN/0.10% TEA in water at 1 mL/min. This method affords retention time of [^18^F]FBAT which was 18.1–18.9 min. Fractions of [^18^F]FBAT and metabolites were calculated for each sample based on the area under each peak. The radioactivity concentration in the whole blood and plasma was assayed using a gamma counter (Cobra, Packard, CT).

### 2.10. In Vivo PET Imaging

Mice (*n* = 30) were anesthetized with 2% isoflurane (in oxygen) and injected with [^18^F]FBAT (11.1 MBq; 0.3 mCi) via the tail vein. Dynamic PET images were obtained in fully three-dimensional list mode for 30 min using a small animal SuperArgus 2r PET system (SEDECAL, Madrid, Spain) or 7T PETMR Inline (Bruker, Rheinstetten, Germany; energy window, 350-650 keV; timing window, 6 ns). Images were acquired every 10 s for 12 images, 60 s for three images, 300 s for three images, or 600 s for four images. The anatomical structure of the brain was imaged using T2 MRI. The MRI sequences included 0.5 mm thick T2 Turbo RARE high-resolution images (TR = 3000 ms, TE, 46 ms, matrix, 512 × 256, average, 40, slice number, 30, field of view, 20 × 10 mm).

### 2.11. In Vivo Blocking Study

After determining the optimal imaging time point for [^18^F]FBAT, we performed a blocking study using a selective iNOS inhibitor to assess the specificity of the tracer for iNOS. Separate 7T PET/MR studies with [^18^F]FBAT were performed for mice (*n* = 6) administered a bolus of aminoguanidine (50 mg/kg *i.p*.) 30 min prior to administration of [^18^F]FBAT. The time course of [^18^F]FBAT radioactivity in the brain after administration of the iNOS inhibitor was assessed and compared to that of the mice in the LPS 3 h group.

### 2.12. PET Imaging Analysis

Images were reconstructed by the Fourier rebinning algorithm and two-dimensional filtered back projection using a ramp filter with a cutoff at Nyquist. The regional radioactivity concentration (kBq/cc) of [^18^F]FBAT was estimated from the mean pixel values within the region of interest (ROI) corresponding to MR images of various organs and regions of the brain. ROI for carotid artery, heart muscle, lung, liver, spleen, and kidney were defined and time activity curves (TACs) generated. TACs for whole brain, cortex, cerebellum, and brainstem were also determined as described above.

The concentration of radioactivity (kBq/cc, *μ*Ci/cc) was converted to standardized uptake value (SUV), and the mean and standard deviation (SD) of radiotracer accumulation values were calculated for different organs and regions of the brain. Data were analyzed with PMOD 4.0 software (PMOD Technologies Ltd., Zurich, Switzerland).

### 2.13. Graphic Logan Graphical Analyses

The dynamic PET imaging data were analyzed using Logan's model-independent graphical analysis [22] to assess whether [^18^F]FBAT PET/MR could be used to detect differences in expression of iNOS in brain regions (*C*_bra_(*t*)). Cardiac blood TACs (*C*_ref_(*t*)) were used as the reference tissue. The slope of the linear portion of the Logan plot represents the total distribution (*V*_t_). Then, the slope of the linear portion of the plot can be calculated using

(1) $$\begin{array}{c} \frac{\int_{0}^{t}C_{\text{reference}}\left(t\right)dt}{C_{\text{reference}}\left(\text{T}\right)}=\text{DVR}\frac{\int_{0}^{t}C_{\text{brain region}}\left(t\right)dt}{C_{\text{reference}}\left(T\right)}+C. \end{array}$$

### 2.14. Pharmacokinetic Two-Compartment Modeling

A time activity curve (TAC) for a carotid artery region of interest was used to determine the dynamic PET image-derived [^18^F]FBAT input function in uncorrected blood plasma. The corresponding TACs for [^18^F]FBAT were derived by applying images from mice administered [^18^F]FBAT to the image-derived plasma TAC. Model parameters were estimated for influx constant *k*1 (mL/cm^3^/min^−1^), efflux (*k*2) (min^−1^) rate of radioligand diffusion between plasma and brain compartment. Exchange between compartments *k*3 (min^−1^) and *k*4 (min^−1^) was also estimated. The net influx constant, *K*_i_ (min^−1^), parameter that describes the rate of binding to the iNOS was calculated as

(2) $$\begin{array}{c} {K_{\text{i}}}_{\text{FBAT}}=\frac{k1_{\text{FBAT}}\times k3_{\text{FBAT}}}{k2_{\text{FBAT}}+k3_{\text{FBAT}}}. \end{array}$$

Compartmental modeling, pharmacokinetic analyses, and generation of pixel-by-pixel parametric images were accomplished using PMOD 4.0 software (PMOD Technologies Ltd., Zurich, Switzerland).

### 2.15. Immunohistochemistry

After imaging, mice were terminally anesthetized with ketamine/xylazine and perfused with 4% paraformaldehyde, and the brains were dissected, postfixed overnight in 4% paraformaldehyde at 4°C. Five-micrometer thick paraffin-embedded brain tissues were deparaffinized, rehydrated, microwaved in 10 mM citrate buffer (pH 6.0) at 100°C for 10 min for antigen retrieval, washed, incubated in 3% hydrogen peroxidase for 15 min at RT to inhibit endogenous peroxidases, and blocked in blocking solution for 60 min at RT.

Sections were incubated with primary NOS2/NOS1 antibodies (Cat# MAB 1627; Abnova, Walnut, CA, USA) diluted 1 : 100 overnight at 4°C, developed using the Vectastain Elite kit (Vector Laboratories, Burlingame, CA) following the manufacturer's instructions, and examined using an AxioScope A1 microscope (Zeiss, Oberkochen, Germany) equipped with an Axiocam 512 color digital camera (Zeiss).

The immunohistochemistry images were converted into 8-bit grayscale images in the [0–255] range. Immunostaining intensity was manually measured in the region of interest (ROI). The median (25%, 75% interquartile range) percentage score for each group (control, LPS 3 h, and LPS 24 h) was calculated as the sum of the individual number of slides within the group.

### 2.16. Statistics

Data are presented as the mean ± SD values for each group, and were compared using one-way ANOVA and the post hoc Bonferroni test or unpaired *t-*tests with Welch's correction using GraphPad Prism 8 (GraphPad Software, La Jolla, CA, USA). *P* < 0.05 was considered significant.

## 3. Results

### 3.1. Chemistry

The preparation of FBAT precursor and authentic product [^19^F]FBAT is shown in Schemes 1 and 2. Briefly, the start material 4-piperidone was protected with ethylene glycol and converted to compound 7 then reacted with 4-iodo- or 4-fluorobenzoyl chloride via acylation to yield compound 1 and compound 8 with excellent yields, respectively. Compound 6 was obtained from 2-amino-3,6-difluorobenzonitrile with hydroxylamine condensation and followed by Raney Ni reduction. The quinazolinamines, compound 3 and FBAT, were prepared from the condensation of compound 6 with the hydrolysis products of 1 and 8, respectively. Then, compound 3 was treated with Boc anhydride and modified by boronic ester via catalytic palladium coupling to yield FBAT precursor for radiofluorination. The authentic FBAT can be applied for analytic HPLC quality control.

### 3.2. Automated Radiosynthesis and Characterization of [^18^F]FBAT

We successfully completed five runs of n.c.a. [^18^F]FBAT preparation using a robotic system. We found that the optimization of precursor/Cu(OTf)_2_ ratio was 1.46 and temperature was 110°C (Figures 1(a) and 1(b)). On average, a typical run produced 592-851 MBq (16–23 mCi) of [^18^F]FBAT at a radiochemical yield of 2.2-3.1% (uncorrected for decay). A silica plus Sep-Pak cartridge was used to trap the unreacted [^18^F]fluoride. The total synthesis time was 200 min. The retention time (*t*_R_) of [^18^F]FBAT in semipreparative HPLC was 10.07 min (Suppl. Fig. 4A). The radioactive product was coinjected with an authentic FBAT standard. The retention time (*t*_R_) of [^18^F]FBAT (Suppl. Fig. 4B) in HPLC analysis was 14.38 min, which was consistent with that of authentic FBAT (Suppl. Fig. 4C). Molar activity (at the end of synthesis) ranged from 125 to 137 GBq/*μ*mol. The radiochemical purity determined by HPLC was greater than 99%. The total volume of the purified product was 2.0 mL, and the pH was 6.0. Acetonitrile and trimethylamine were not detected in the final product. The Log *P* of FBAT measured between 1-octanol and phosphate buffer at pH 7.4 was 1.40 ± 0.27, which is a moderate value suitable for a brain imaging agent.

### 3.3. LPS Induces NO Production in a Dose-Dependent Manner

**Figure 2:**
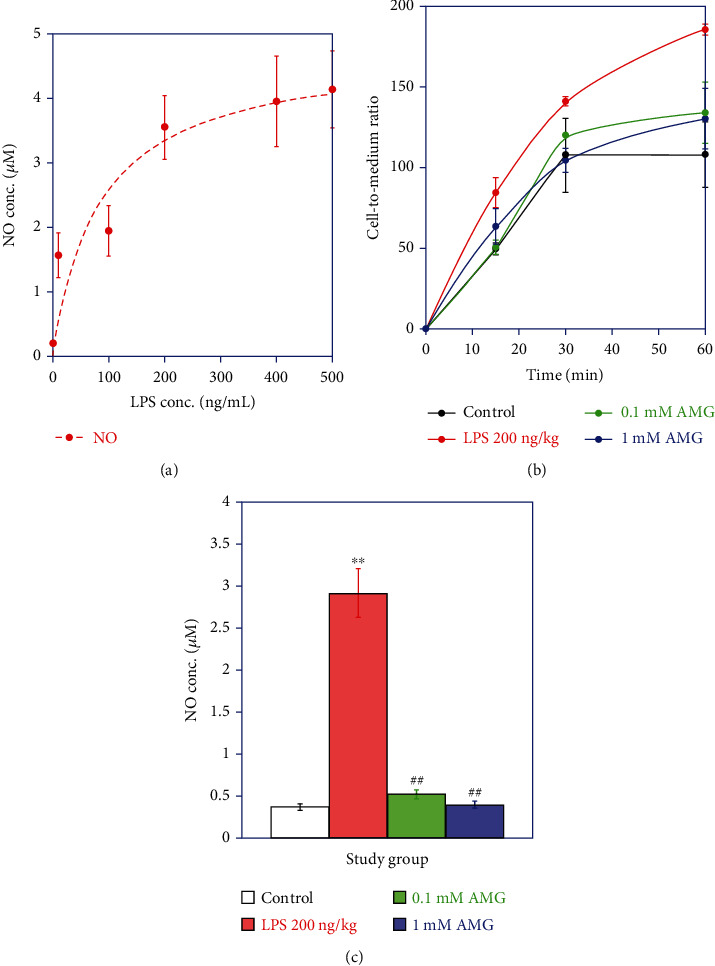
The LPS- and LPS+ AMG-induced iNOS expression and NO production. (a) NO production in the presence of different concentrations of LPS. (b) Time-dependent uptake of [^18^F]FBAT. Control BV2 cells (black line), LPS-induced BV2 cell (red line), and LPS-induced BV2 cell pretreated with 1 mM (blue line) or 0.1 mM aminoguanidine (green line). (c) NO production in control cell, cell treated with LPS, and cell pretreated with 1 mM or 0.1 mM aminoguanidine before LPS treatment. Data are mean ± SD (*n* = 3 per group). ^∗∗^*P* < 0.005 compared to controls; ^##^*P* < 0.005 compared to the LPS 3 h group.

LPS stimulation of NO release has been well established as an in vitro model of microglia activation. As shown in Figure 2(a), NO production concentration dependently increased in BV2 cells treated with LPS.

### 3.4. Preferential Accumulation of [^18^F]FBAT in BV2 Cells after LPS Induction

All groups exhibited rapid uptake of [^18^F]FBAT during the initial phase (first 15 min). Thereafter, the accumulation of [^18^F]FBAT reached a plateau in control BV2 cells (black line) around a cells-per-medium concentration ratio of 100. In contrast, in LPS-induced BV2 cells (red line), [^18^F]FBAT accumulation continued to increase up to 1 h and thereafter reached a plateau at a cells-per-medium concentration ratio of 180–200. [^18^F]FBAT accumulation at 60 min was more than 1.7-fold higher in LPS-induced BV2 cells than control BV2 cells (Figure 2(b)). Pretreatment of the BV2 cells with 0.1 mM or 1 mM AMG for 1 h significantly reduced the LPS-induced [^18^F]FBAT accumulation (*P* < 0.05) (Figure 2(b), green or blue lines).

Furthermore, we analyzed NO production in murine microglial BV2 cells after [^18^F]FBAT cell uptake study. LPS significantly increased NO production compared to control cells. LPS-induced NO production was completely abolished by pretreatment with 0.1 or 1 mM of the iNOS inhibitor aminoguanidine (Figure 2(c)).

### 3.5. In Vivo Metabolism Study in Plasma

**Figure 3:**
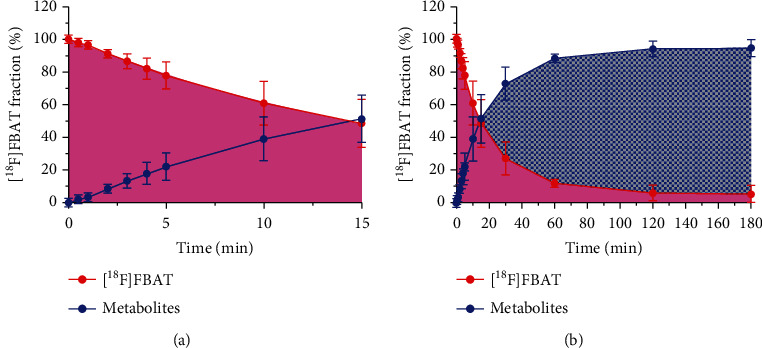
Serial PET/MR images of temporal dynamics of [^18^F]FBAT-derived radioactivity distribution in mice: control and LPS groups (5 mg/kg, i.p.). Representative PET/MR images are provided in axial planes with color coding based on the range of [^18^F]FBAT-derived radioactivity accumulation between 0 and 5 SUV to maximize the visualization of different organs.

Metabolism study has shown a rapid decrease of the parent fraction in plasma; the parent compound accounted for 77.98% ± 8.36 in plasma at the first 5 minutes (Figure 3(a)). Roughly 51.41% ± 14.66 and 94.02% ± 4.75 of the metabolites were observed 15 min and 120 min after radiotracer injection, respectively (Figure 3(b)).

### 3.6. Quantitative Whole-Body Biodistribution in Mice

**Figure 4:**
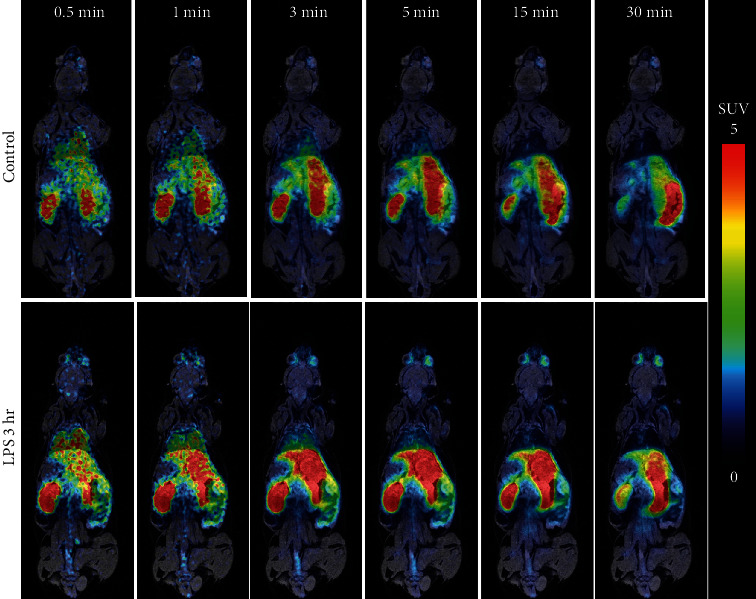
Time activity curves of [^18^F]FBAT-derived radioactivity (SUV) in blood and different organs in the control and LPS groups (5 mg/kg, i.p.). Data are mean ± SD (*n* = 3 per group).

In vivo dynamic PET/MR imaging was performed in six mice injected with 5 mg/kg LPS and six control mice after administration of [^18^F]FBAT. Generally, PET/MR imaging revealed rapid accumulation of [^18^F]FBAT in LPS-injected mice and the control group (Figure 4). No accumulation of [^18^F]FBAT-derived radioactivity was detected in the skeletal structures up to 30 min postinjection of [^18^F]FBAT. [^18^F]FBAT uptake by Harderian glands was not markedly increased and influenced the uptake of brain regions.

**Figure 5:**
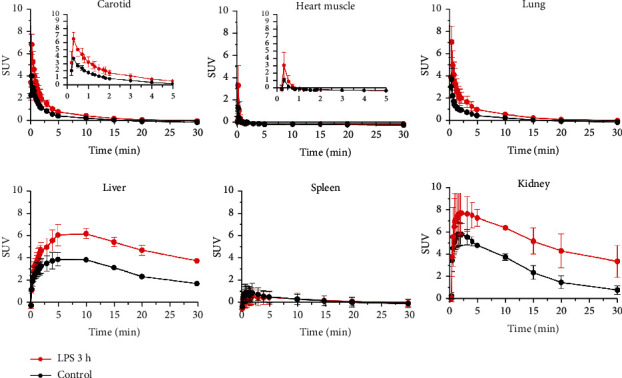
Pharmacokinetics of [^18^F]FBAT after i.v. administration. (a) Time course of [^18^F]FBAT (parent) and metabolite fraction in blood plasma, expressed as % fraction. (b) The same data as (a), but over a different time scale (0-5 min). Data are mean ± SD (*n* = 3).

[^18^F]FBAT exhibited biexponential blood (carotid) clearance kinetics after i.v. injection (Figure 5). The concentration of radioactivity remaining in the blood pool was 0.298 ± 0.019 SUV at 30 min postinjection. This pattern of hepatobiliary clearance was followed by a fast increased radioactivity in the blood and liver and subsequent clearance by the kidneys. The pattern of renal clearance was followed by a rapid increase in radioactivity in the blood and liver and subsequent clearance by the kidneys in the first 5 minutes. Rapid accumulation of [^18^F]FBAT in the lungs, peaking at 1-2 minutes postinjection, was also observed. The concentration of radioactivity in heart muscle peaked in the first 1 min after injection and then gradually decreased over time (Figure 5).

### 3.7. In Vivo PET Imaging with [^18^F]FBAT Enables Discrimination of LPS-Induced iNOS Expression

**Figure 6:**
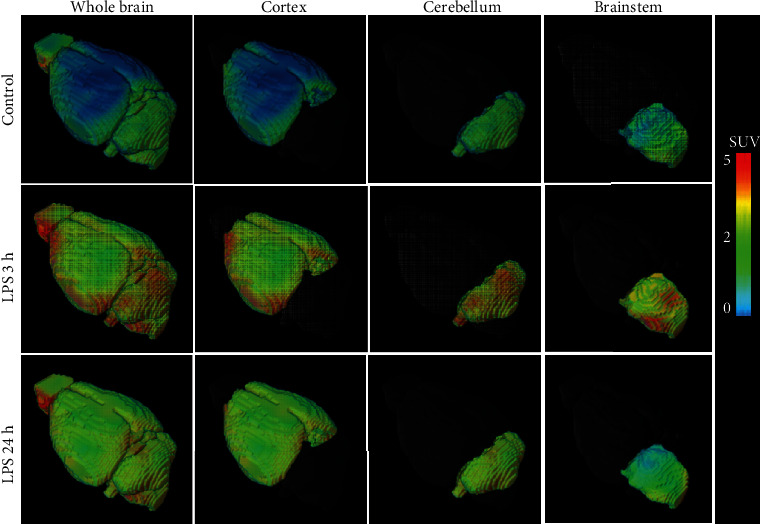
3D PET images of FBAT at 3 h and 24 h postinduction of LPS. Representative 3D PET images of LPS-induced inflammation at 3 or 24 h post i.v. injection of LPS mice. Images were summed from 0 to 30 min after injection of [^18^F]FBAT. The accumulated radioactivity is expressed in the unit of SUV (*n* = 6 per group). PET Images are color coded to SUV (*n* = 6 per group).

**Figure 7:**
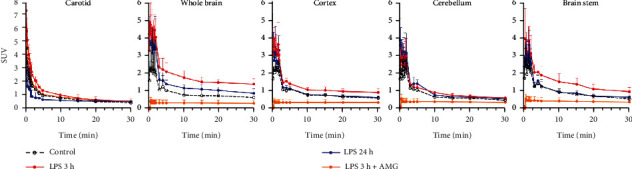
Time activity curves for each region of interest within the brain over 30 min of dynamic PET images. PET imaging showed rapid accumulation of [^18^F]FBAT in control or LPS-induced mice. The level of [^18^F]FBAT accumulation was significant in the whole brain, cortex, and brainstem at 3 h postinjection of LPS whereas no different accumulation after 24 h LPS induction. In contrast to the whole brain and cortex, there was similar [^18^F]FBAT accumulation in the cerebellum before and after treatment with LPS. The accumulated radioactivity is expressed in the unit of SUV. Data are mean ± SD of the voxel values within each ROI (*n* = 6 per group).

*In vivo* PET with [^18^F]FBAT demonstrated heterogeneously increased, transient accumulation of [^18^F]FBAT radioactivity in LPS mice (Figure 6; *n* = 6 per group). Clearance of [^18^F]FBAT from their circulation exhibited a biexponential kinetics with half-lives of 1.05 ± 0.58 min and 24.9 ± 7.46 min, respectively (Figure 7, carotid). At 30 min post i.v. injection, the level of [^18^F]FBAT in blood was 0.30 ± 0.06 SUV (standardized uptake value), determined from the mean pixel activity within the ROI placed over the carotid region. The time activity curve of carotid as an input function was applied for Logan plot to estimate total volume distribution of [^18^F]FBAT *or* pharmacokinetic two-compartment model. One of the aims of the study was to preliminarily measure and analyze [^18^F]FBAT accumulation in the three major regions: cortex, cerebellum, and brainstem. Whole brain was used as an average uptake per voxel in all regions. PET imaging revealed rapidly cross BBB and accumulation of [^18^F]FBAT in the whole brain and, to a lesser degree, in the cortex, cerebellum, and brainstem. After LPS induction, the radioactivity of [^18^F]FBAT in the whole brain, cortex, cerebellum, and brainstem reflected the concentration of [^18^F]FBAT in the blood pool and was surprisingly high at 3 h postinjection of LPS. Radioactive accumulation of [^18^F]FBAT was 1.43 ± 0.11 SUV in the whole brain, 0.90 ± 0.07 SUV in the cortex, 0.60 ± 0.03 SUV in the cerebellum, and 0.98 ± 0.09 SUV in the brainstem (Figure 7; 3 h-LPS red solid line). At 24 h after LPS stimulation and 30 min after the tracer was injected, [^18^F]FBAT accumulation reached 0.90 ± 0.13 SUV in the whole brain, 0.62 ± 0.02 SUV in the cortex, 0.54 ± 0.02 SUV in the cerebellum, and 0.67 ± 0.07 SUV in the brainstem (Figure 7; 24 h-LPS blue solid lines).

**Figure 8:**
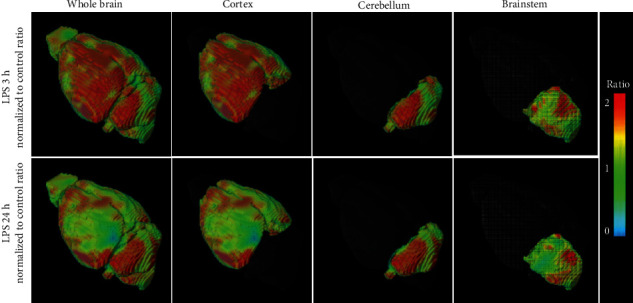
3D parametric SUV ratio images of [^18^F]FBAT at 3 and 24 h postinduction of LPS. PET images were summed from 0 to 30 min after injection of [^18^F]FBAT. The ratio of accumulated radioactivity between control and LPS 3 h was marked in the whole brain, cortex, cerebellum, and brainstem whereas no such difference in the brain at control and 24 h postinjection of LPS. PET images are color coded to SUV ratio (*n* = 6 per group).

**Figure 9:**
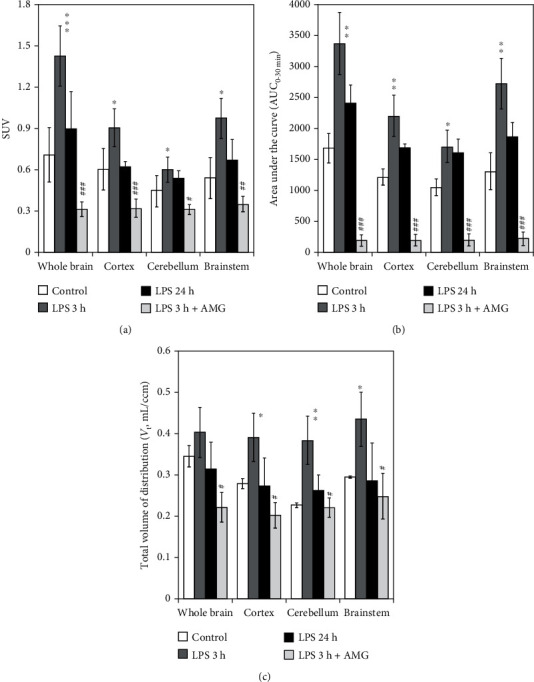
Standard uptake values (SUV), area under the curve (AUC_0-30min_), or volume distribution (*V*_t_) of [^18^F]FBAT. (a) Accumulated radioactivity is expressed in SUV at 20-30 min postinjection of [^18^F]FBAT. (b) Dynamitic time activity curves (0-30 min) were used to determine the AUC_0-30min_ for all scans. (c) Volume distribution (*V*_t_, mL/cm^3^) of [^18^F]FBAT determined by Logan model-independent graphical analysis. Data are mean ± SD; ^∗^*P* < 0.05, ^∗∗^*P* < 0.005, and ^∗∗∗^*P* < 0.001 compared to the controls; ^#^*P* < 0.05, ^##^*P* < 0.005, and ^###^*P* < 0.001 compared to the LPS 3 h group.

The parametric images were used to determine the standardized uptake value ratios (SURs) after normalization to the control group (Figure 8). SURs (SUV_LPS-3h_ to SUV_con_) were 2.16 ± 0.18 (^∗^*P* < 0.05) in the whole brain, 1.53 ± 0.05 (^∗^*P* < 0.05) in the cortex, 1.41 ± 0.21 (^∗^*P* < 0.05) in the cerebellum, and 1.91 ± 0.12 (^∗^*P* < 0.05) in the brainstem. The quantitative measures of [^18^F]FBAT accumulation at 30 min post *i.v*. injection of [^18^F]FBAT at various time points in the LPS-induced model are summarized in Figure 9(a) and Table 1.

Mouse brain exposure to [^18^F]FBAT was estimated in all conditions (control or LPS-induced groups) using the mean area under the curve of the tissue radioactivity from 0 to 30 min (AUC; SUV·min). Integrated activity (0-30 min) showed more [^18^F]FBAT retention in the LPS 3 h group in comparison to control or LPS 24 h mice (Figure 9(b) and Table 2).

### 3.8. Neuroinflammation Appears Early on PET iNOS Imaging

In model-independent Logan graphical analysis of dynamic PET imaging data using blood as the reference tissue devoid of iNOS protein expression, the average volume distribution (*V*_t_, mL/cm^3^) for [^18^F]FBAT in brain regions was 0.40 ± 0.02 (Figure 9(b)). In control animals, *V*_t_ of [^18^F]FBAT in the brain regions was 0.29 ± 0.05. The differences in *V*_t_ between the LPS and control groups were statistically significant in the cortex, cerebellum, and brainstem (Figure 9(c) and Table 3).

Additional to using Logan graphical analysis to calculate volume distribution (*V*_t_, mL/cm^3^), the net influx rate constant (*K*_i_, min^−1^) was also calculated using two-tissue compartment model. The whole brain *K*_i_ of [^18^F]FBAT (0.007 ± 0.003 min^−1^) in the LPS group was significantly higher than that of controls (4.45*E* − 06 ± 2.01*E* − 06 min^−1^). The influx (*k*1, mL/cm^3^/min) and efflux (*k*2, 1/min) rate constants of [^18^F]FBAT in the whole brain were no difference between the control and LPS groups which are consistent with the moderate lipophilicity of the radiotracer. The [^18^F]FBAT *k*1, *k*2, and *k*3 rate constants and *K*_i_ for various brain structures are presented in Table 4.

### 3.9. Aminoguanidine Significantly Inhibits the Accumulation of [^18^F]FBAT in iNOS-Expressing Regions of the Brain of LPS-Induced Mice

*In vivo* dynamic PET/MR imaging revealed pretreatment with aminoguanidine (50 mg/kg) 30 min before LPS induction significantly reduced [^18^F]FBAT accumulation by decreasing iNOS expression, with corresponding SUVs of 0.31 ± 0.08, 0.32 ± 0.09, 0.31 ± 0.09, and 0.35 ± 0.10 in the whole brain, cortex, cerebellum, and brainstem, respectively (Figures 6, 7, and 9(a)). Logan graphical analyses showed pretreatment with aminoguanidine significantly reduced the AUC and *V*_t_ values of the whole brain, cortex, cerebellum, and brainstem (^###^*P* < 0.001 and ^#^*P* < 0.05, respectively; Figures 9(b) and 9(c)). The quantitative measures of [^18^F]FBAT accumulation 30 min after *i.v*. administration of [^18^F]FBAT with or without the iNOS inhibitor are summarized in Tables 2 and 3.

### 3.10. Quantitative Immunostaining Supports the Results of In Vivo PET/MR Imaging with [^18^F]FBAT

**Figure 10:**
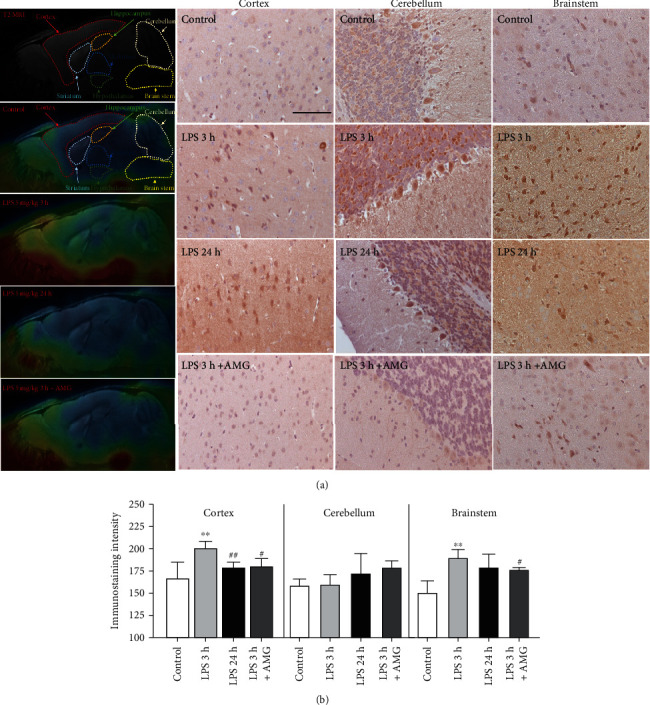
Comparative analysis of [^18^F]FBAT PET/MRI and immunohistochemical (IHC) staining for iNOS in the cortex and cerebellum. (a) Representative sagittal PET/MR images obtained 30 min postinjection of [^18^F]FBAT in control, LPS-induced mice, and LPS-induced mice pretreated with aminoguanidine. [^18^F]FBAT accumulation significantly decreased in all brain regions after administration of aminoguanidine. Color coding of the images is set to maximize the visualization of [^18^F]FBAT accumulation in each projection. (b) Validation of [^18^F]FBAT PET using histopathological analyses of iNOS expression in the brain at 3 h or 24 h postinjection of LPS. Stronger iNOS (+) imaging is representative of the positive staining results obtained in the cortex, cerebellum, and brainstem in the 3 h LPS-induced mice compared to the 24 h LPS-induced group and controls. Pretreatment with aminoguanidine significantly reduced iNOS immunoactivity in the cortex and brainstem, but not in the cerebellum. Scale bar = 100 *μ*m. Data are mean ± SD; *n* = 3 per group; ^∗∗^*P* < 0.01 compared to the control group; ^#^*P* < 0.05 and ^##^*P* < 0.005 compared to the LPS 3 h group.

To validate the ability of PET/MRI to specifically detect iNOS expression and quantify regional-specific uptake of [^18^F]FBAT, the mice were sacrificed after imaging and the brains were excised for histologic analyses (*n* = 3 per group). Immunostaining for iNOS (Figure 10(a)) confirmed the region-specific localization of iNOS in the brain observed in the [^18^F]FBAT PET/MRI images (Figure 10(a) white squares).

Quantitative analysis of the immunostained sections confirmed iNOS immunoactivity preferentially accumulated in viable portions of the cerebellum and cortex of mice with LPS-induced inflammatory responses (in the 3 h LPS group; Figure 10(b)). Moreover, higher iNOS immunoactivity was observed in the cortex, granular layer, and Purkinje cells in the cerebellum at 3 h postinjection of [^18^F]FBAT. In the cortex, iNOS immunoactivity was higher at 3 h after injection of LPS than 24 h after injection of LPS (^∗∗^*P* < 0.01), whereas iNOS immunoactivity in the cerebellum was not significantly different between the LPS 3 h and 24 h groups (Figure 10(b)). Pretreatment with aminoguanidine significantly reduced LPS-induced iNOS immunoreactivity in the cortex (^#^*P* < 0.05), but not in the cerebellum, compared to the LPS 3 h group.

## 4. Discussion

^18^F-labeled aromatics are the most commonly prepared using S_N_Ar reactions [23, 24]. These reactions typically require high temperatures (often >150°C) and are restricted to electron-deficient substrates. In this study, we initially used a nitro compound as a precursor [15], though the radiochemical yield (<0.1%, uncorrected for decay) and molar activity (2 GBq/*μ*mol) were very poor. It would be very difficult to carry out radiofluorination by substituting nitro group on the benzene ring if there were no electron-withdrawing groups in the nitrobenzene ring. Recent advances have expanded the scope of nucleophilic aromatic radiofluorination by using triarylsulfonium diarylselenone [25] [26] or iodonium ylide precursors [27–29]. The Sanford group previously reported Cu-mediated fluorination of aryl trifluoroborates, arylboronate esters, and arylboronic acids with KF [30] which provides an alternative way to perform radiofluorination of benzene ring with the lack of electron-withdrawing groups and obtain an acceptable yield. Copper-mediated aromatic nucleophilic radiofluorination was used to radiosynthesize [^18^F]FBAT by [31] a boron ester 2-N-protected precursor. Pyridine is essential for copper-mediated radiolabeling. The radiochemical yield increased when we increased the pyridine/Cu(II)(OTf)_2_ molar ratio from 15 to 25, in agreement with a previous study [32]. However, future studies will explore the potential strategies for reducing the time (200 min) and cost of radiosynthesis for [^18^F]FBAT.

Radiolabeled [^18^F]F-NOS has been previously investigated and demonstrated that iNOS activity correlated significantly with lung tissue measurements in healthy volunteers and in myocardial tissue of orthotopic heart transplant patients undergoing surveillance for rejection [33, 34]. Expression of iNOS in activated macrophages was assessed *in vivo* using [^18^F]F-6-(2-fluoropropyl)-4-methyl-pyridin-2-amine, both in a LPS-induced lung inflammation of mouse model and endotoxin-induced lung inflammation in healthy volunteers. Significantly higher [^18^F]F-6-(2-fluoropropyl)-4-methyl-pyridin-2-amine uptake was reported in the lungs of mice treated with LPS than control mice [15]. However, no studies of neuroinflammation in animal or humans using this approach have yet been reported.

To our knowledge, this is the first report of [^18^F]FBAT synthesis and the first histologically validated study of [^18^F]FBAT for *in vitro* and *in vivo* PET imaging. We demonstrated that LPS-induced microglial BV2 cells, which are the most frequently used *in vitro* model of primary microglia [35], exhibited significantly higher accumulation of [^18^F]FBAT than control cells. Preliminary semiquantitative measurements (metabolite-uncorrected) of [^18^F]FBAT-PET demonstrated rapid, high accumulation of [^18^F]FBAT in the brains of mice with LPS-induced brain inflammation. We also observed significantly higher accumulation of [^18^F]FBAT in the brain at 3 h after LPS stimulation than at 24 h after LPS stimulation.

Our metabolism study showed a rapid decrease of the parent fraction in plasma where roughly 50% of the metabolites was detected at 15 min postinjection of [^18^F]FBAT. However, most of the iNOS radioligands available are also known to show similar metabolism. For example, [^11^C]L-NAME revealed that at 10 min postinjection 65% of the radioactivity was [^11^C]methanol (metabolite) in dog [36]; within 5 min postinjection, only 40.3% of the activity in the blood was parent [^18^F]9 [16]. This metabolism in the plasma may be a disadvantage in terms of [^18^F]FBAT use since it makes its quantification more difficult. Nevertheless, the contribution of the major metabolites of [^18^F]FBAT circulating in blood which led to accumulation of [^18^F]FBAT-derived radioactivity in the brain needs to be accounted for in future investigations.

The results of *in vivo* biodistribution studies showed that the highest uptake in both groups was observed in the liver and kidneys, which are likely to be the major metabolic and/or excretory sites for [^18^F]FBAT. The rapid whole-body redistribution and fast renal clearance of [^18^F]FBAT from circulation make this radiotracer especially suitable for imaging of the chest/abdominal area, specifically of the lung. No significant accumulation of [^18^F]FBAT-derived radioactivity was detected in the skeletal structures up to 30 min postinjection of [^18^F]FBAT, which suggests that the radiotracer was not catabolized or defluorinated *in vivo*. Our results of whole-body biodistribution of [^18^F]FBAT-derived radioactivity were in agreement with iNOS distribution in organs in male BLAB/c mice that reported iNOS mRNA and protein expression 6 h after LPS stimulation was observed in many organs. Among them, the highest iNOS expression was in the lungs followed by that in the kidneys, heart, gut, and liver [37].

A similar pattern of radioactivity biodistribution was previously reported for S-[^11^C]methylisothiourea ([^11^C]MITU) and S-(2-[^18^F]fluoroethyl)isothiourea ([^18^F]FEITU) in mature female Sprague-Dawley rats in control and pretreated with 10-20 mg/kg LPS, 6 h before tracer injection of radiotracers [12], and also similar with that in mature male C57BL/6 mice with intravenous injection with LPS (10 mg/kg) 6 h prior to the tracer injection to induce iNOS expression [16].

In this initial evaluation, we used AUC and *V*_t_ of [^18^F]FBAT to quantify [^18^F]FBAT accumulation in the brain from the PET images. The AUC_0-30min_ and *V*_t_ values of [^18^F]FBAT in the whole brain were averagely 1.9 ± 0.21- and 1.4 ± 0.22-fold lower in control mice than in the LPS 3 h group. After treatment with selective iNOS inhibitor, AUC_0-30min_ and *V*_t_ values showed a statistically significant reduction in AUC and *V*_t_ of [^18^F]FBAT in the LPS group (*P* < 0.001 and *P* < 0.05, respectively).

Further studies such as coupling imaging of PET [^18^F]FBAT and dynamic contrast-enhanced MRI (DCE-MRI) assess dose dependence of LPS on BBB permeability/disruption in rodent model or neuroinflammation produced by intracerebral injection of LPS.

The higher resolution 7T PET/MR system was used to enable more detailed assessment of the heterogeneous accumulation of [^18^F]FBAT radioactivity. The PET/MR results indicated that the radioactivity of [^18^F]FBAT correlated with the levels of iNOS expression induced by cellular inflammatory responses in the brain observed using quantitative immunohistochemistry (QIHC). Pretreatment of animals with the selective iNOS inhibitor aminoguanidine significantly decreased [^18^F]FBAT accumulation in all structures of the brain, consistent with the QIHC studies.

In comparison with previous reports of iNOS imaging agents [11–16], the results of our *in vivo* PET imaging studies with [^18^F]FBAT in mouse brain expressing different levels of iNOS after LPS inducement are more selective. Predominant accumulation of [^18^F]FBAT in iNOS-expressing brain regions could be implied to be responsive to therapy with selective iNOS inhibitor (e.g., aminoguanidine) [38].

The LPS animal model of neuroinflammation used in this study has several essential advantages, including technical ease and high reproducibility, particularly with respect to the magnitude of the inflammatory response elicited. Shortly after administration of LPS, high levels of proinflammatory cytokines are released and lead to rapid development of systemic inflammatory response syndrome (SIRS) and subsequent dose-dependent mortality [39, 40]. Thus, a well-characterized method is needed to monitor the inflammatory reactions *in vivo* after LPS treatment. Hou et al. [41] used [^18^F]-ROStrace PET imaging targeted to superoxide and reported the level of neuroinflammation induced by LPS (5 mg/kg) was strongly associated with the severity of the pain and distress scores [42].

Another critical issue that needs to be addressed is whether [^18^F]FBAT in the systemic circulation crosses the BBB and directly activates cells within the CNS. Based on the current results, we consider that [^18^F]FBAT crosses the BBB and cell membranes bidirectionally by nonfacilitated diffusion, due to its physicochemical characteristics (Log *P* 1.40). Therefore, the rate constants of [^18^F]FBAT influx (*k*1_FBAT_) and efflux (*k*2_FBAT_) across the BBB and cell membranes are assumed to be high and not rate limiting. The critical issue of whether LPS actually disrupts the BBB and results in overestimation of [^18^F]FBAT accumulation in the brain can be answered using the pharmacokinetic two-compartment model. Based on our preliminary evaluation, [^18^F]FBAT influx (*k*1_FBAT_) and efflux (*k*2_FBAT_) were similar with or without LPS induction; however, *k*3 was significantly different between LPS-injected and control mice. This suggests that the higher [^18^F]FBAT *V*_t_ observed in the brain of LPS-injected mice is most likely due to increased expression of iNOS in the brain parenchyma. Also, due to the low brain uptake of the tracer (1 to 2 SUV), cerebral passage of [^18^F]FBAT does not depend on cerebral blood flow. Thus, *V*_t_ represents the most robust parameter for quantifying [^18^F]FBAT uptake and is not affected by changes in cerebral blood flow. Nevertheless, further metabolite analysis of [^18^F]FBAT in the brain and knowledge of the dose-response relationship between LPS and [^18^F]FBAT accumulation in the brain are required to validate the implications of BBB disruption by LPS.

## 5. Conclusion

An automated robotic method was established for radiosynthesis of [^18^F]FBAT from a boron ester precursor in a hot cell. A HPLC method was employed to purify the product. The radiochemical purity of [^18^F]FBAT was greater than 99%, and the corrected radiochemical yield was about 2.2-3.1% (EOS). Preliminary *in vitro* and *in vivo* evaluations demonstrated [^18^F]FBAT could potentially be used to detect iNOS activity and expression in LPS-induced neuroinflammation. Further investigations, including optimization of the radiochemical yield and detailed brain region-specific distribution in animal PET/MRI imaging research applications, are currently underway in our laboratory.

## Figures and Tables

**Table 1:** Comparison of PET standardized uptake value (SUV) and standardized uptake value ratio (SUR) of [^18^F]FBAT in control *vs.* LPS mice at 3 or 24 h post *i.v.* injection of LPS (SUR_LPS_ to SUV_con_).

SUV	Control	LPS 3 h	LPS 24 h	LPS 3 h+AMG
Whole brain	0.709 ± 0.200	1.428 ± 0.410^∗∗∗^	0.900 ± 0.270	0.312 ± 0.055^###^
*SUR*	1.000	2.163 ± 0.177^∗^	1.351 ± 0.451	N/A
Cortex	0.604 ± 0.150	0.904 ± 0.251^∗^	0.620 ± 0.041	0.320 ± 0.063^###^
*SUR*	1.000	1.525 ± 0.046^∗^	0.970 ± 0.303	N/A
Cerebellum	0.448 ± 0.115	0.600 ± 0.066^∗^	0.542 ± 0.052	0.310 ± 0.037^#^
*SUR*	1.000	1.406 ± 0.211^∗^	1.233 ± 0.112	N/A
Brainstem	0.540 ± 0.153	0.976 ± 0.244^∗^	0.671 ± 0.148	0.350 ± 0.055^##^
*SUR*	1.000	1.906 ± 0.116^∗^	1.273 ± 0.163	N/A

Data are mean ± SEM; *n* = 6 per group; ^∗^*P* < 0.05, ^∗∗^*P* < 0.005, and ^∗∗∗^*P* < 0.001 compared to controls; ^#^*P* < 0.05, ^##^*P* < 0.005, and ^###^*P* < 0.001 compared to LPS 3 h.

**Table 2:** Comparison of PET mean area under the curve (AUC_0-30min_) of [^18^F]FBAT in control *vs.* LPS mice at 3 or 24 h post *i.v.* injection of LPS.

*AUC*	Control	LPS 3 h	LPS 24 h	LPS 3 h+AMG
Whole brain	1678.6 ± 239.3	3365.4 ± 489.24^∗∗^	2411.5 ± 291.53	193.6 ± 93.84^###^
Cortex	1214.6 ± 133.7	2194.9 ± 250.98^∗∗^	1688.5 ± 61.56	195.5 ± 98.80^###^
Cerebellum	1049.5 ± 132.7	1702.7 ± 125.49^∗^	1614.7 ± 217.86	194.6 ± 95.58^###^
Brainstem	1307.1 ± 296.3	2722.5 ± 652.86^∗∗^	1867.5 ± 227.00	219.8 ± 104.72^###^

Data are mean ± SEM; *n* = 6 per group; ^∗^*P* < 0.05 and ^∗∗^*P* < 0.005 compared to the controls; ^###^*P* < 0.001 compared to the LPS 3 h group.

**Table 3:** Comparison of PET volume distribution (*V*_t_, mL/cm^3^) of [^18^F]FBAT in control *vs.* LPS mice at 3 or 24 h post *i.v.* injection of LPS.

*V*_t_	Control	LPS 3 h	LPS 24 h	LPS 3 h+AMG
Whole brain	0.346 ± 0.012	0.404 ± 0.003	0.274 ± 0.068	0.203 ± 0.032^#^
Cortex	0.278 ± 0.006	0.391 ± 0.020^∗^	0.264 ± 0.036^∗^	0.222 ± 0.024^#^
Cerebellum	0.227 ± 0.001	0.385 ± 0.016^∗∗^	0.288 ± 0.090	0.250 ± 0.055^#^
Brainstem	0.295 ± 0.026	0.435 ± 0.040^∗^	0.315 ± 0.065	0.223 ± 0.036^#^

Data are mean ± SEM; *n* = 6 per group; ^∗^*P* < 0.05 and ^∗∗^*P* < 0.005 compared to the controls; ^#^*P* < 0.05 compared to the LPS 3 h group.

**Table 4:** Pharmacokinetic parameters for [^18^F]FBAT in mouse whole brain.

	*k*1 (min^−1^)	*k*2 (min^−1^)	*k*3 (min^−1^)	*K*_i_ (min^−1^)
Control	0.957 ± 0.250	0.777 ± 0.101	2.86*E* − 05 ± 4.48*E* − 05	4.45*E* − 06 ± 2.01*E* − 06
LPS 5 mg/kg	0.633 ± 0.002	0.608 ± 0.054	0.006 ± 0.003^∗^	0.007 ± 0.003^∗^

Data are mean ± SEM; *n* = 6 per group; ^∗^*P* < 0.05 compared to controls.

## Data Availability

All data generated or analyzed during this study are included in this published article.

## Abbreviations

[^11^C]L-NAME: N-Omega-nitro-L-arginine methyl ester
[^11^C]MITU: *S*-[^11^C]Methylisothiourea
[^18^F]FEITU: S-2-[^18^F]Fluoroethyliso-thiourea
[^18^F]FBAT: (4′-Amino-5′,8′-difluoro-1′H-spiro[piperidine-4,2′-quinazolin]-1-yl)-(4-fluorophenyl)methanone
CNS: Central nervous system
EOS: End of synthesis
eNOS: Endothelial NOS
FFDI: 8-Fluoro-3-(4-fluorophenyl)-3,4-dihydro-1-isoquino-linamine
HPLC: High-performance liquid chromatography
IHC: Immunohistochemistry
IL-1b: Interleukin 1 beta
IL-6: Interleukin-6
IL-8: Interleukin-8
iNOS: Inducible nitric oxide synthase
LPS: Lipopolysaccharide
NCA: No-carrier-added
NO: Nitric oxide
nNOS: Neuronal NOS
PET: Positron emission tomography
QIHC: Quantitative immunohistochemistry
ROI: Region of interest
SD: Standard deviation
SUV: Standardized uptake value
SUR: Standardized uptake value ratio
TNF-*α*: Tumor necrosis factor alpha.

## Supplementary Materials

Supplementary Materials See supplementary material for the structures of PET tracers in iNOS research mentioned in this article, the NMR spectra or LC/MS chromatogram of [^18^F]FBAT precursor or standard, and the retention time (*t*_R_) of [^18^F]FBAT in HPLC analysis. Suppl. Fig. 1: examples of PET tracers in iNOS research. Suppl. Fig. 2: (A) NMR spectra of [18F]FBAT precursor. (B) LC/MS chromatogram of [18F]FBAT precursor. Suppl. Fig. 3: (A) NMR spectra of FBAT standard. (B) LC/MS chromatogram of FBAT standard. Suppl. Fig. 4: (A) the retention time (*t*_R_) of [^18^F]FBAT in semipreparative HPLC was 10.07 min. (B) The retention time (*t*_R_) of [^18^F]FBAT in HPLC analysis was 14.38 min. (C) The retention time (*t*_R_) of authentic FBAT in HPLC analysis was 13.93 min.
