# Supplementary material for: Automated Synthesis and Initial Evaluation of (4′-Amino-5′,8′-difluoro-1′H-spiro[piperidine-4,2′-quinazolin]-1-yl)(4-[18F]fluorophenyl)methanone for PET/MR Imaging of Inducible Nitric Oxide Synthase
Source: Mol Imaging. 2021 Jul 8;2021:9996125. doi: 10.1155/2021/9996125 (PMC8328489; doi:10.1155/2021/9996125)
Supplement: Supplementary Materials — See supplementary material for the structures of PET tracers in iNOS research mentioned in this article, the NMR spectra or LC/MS chromatogram of [18F]FBAT precursor or standard, and the retention time (tR) of [18F]FBAT in HPLC analysis. Suppl. Fig. 1: examples of PET tracers in iNOS research. Suppl. Fig. 2: (A) NMR spectra of [18F]FBAT precursor. (B) LC/MS chromatogram of [18F]FBAT precursor. Suppl. Fig. 3: (A) NMR spectra of FBAT standard. (B) LC/MS chromatogram of FBAT standard. Suppl. Fig. 4: (A) the retention time (tR) of [18F]FBAT in semipreparative HPLC was 10.07 min. (B) The retention time (tR) of [18F]FBAT in HPLC analysis was 14.38 min. (C) The retention time (tR) of authentic FBAT in HPLC analysis was 13.93 min. [file 9996125.f1.zip › Suppl_Fig_4.pdf]

(A)

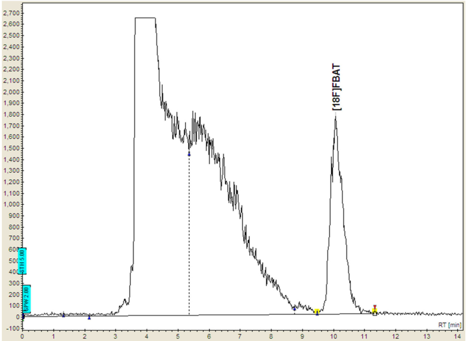

#	Name	Time [Min]	Quantity [% Area	Height [mV]	Area [mV.Min]	Area % [%]
2	UNKNOWN	1.32	0.16	23.3	12.4	0.162
1	UNKNOWN	1.32	0.23	23.3	17.8	0.232
3	UNKNOWN	3.95	50.11	2642.5	3842.4	50.108
4	UNKNOWN	5.55	38.33	1797.0	2938.9	38.326
5	UNKNOWN	8.88	0.50	91.6	38.1	0.497
6	[18F]FBAT	10.07	10.67	1753.2	818.6	10.675
Total			100.00	6330.7	7668.1	100.000

(B)

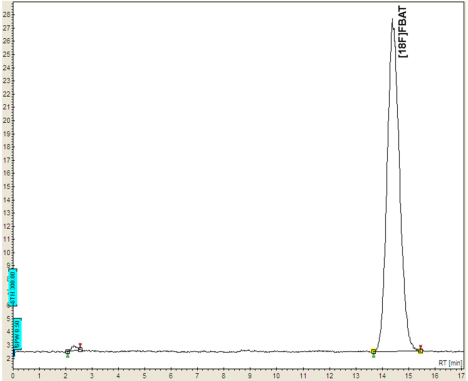

#	Name	Time [Min]	Quantity [% Area	Height [mV]	Area [mV.Min]	Area % [%]
1	UNKNOWN	2.32	0.58	0.4	0.1	0.578
2	[18F]FBAT	14.38	99.42	25.2	13.6	99.422
Total			100.00	25.6	13.7	100.000

(C)

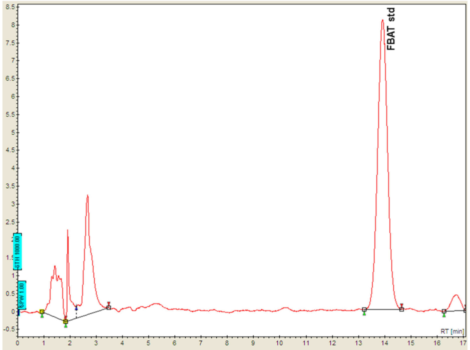

#	Name	Time [Min]	Quantity [% Area	Height [mAU]	Area [mAU.Min]	Area % [%]
1	UNKNOWN	1.42	10.72	1.4	0.6	10.724
2	UNKNOWN	1.91	5.13	2.5	0.3	5.131
3	UNKNOWN	2.67	18.96	3.3	1.1	18.962
4	FBAT std	13.93	62.04	8.1	3.6	62.039
5	UNKNOWN	16.72	3.14	0.4	0.2	3.145
Total			100.00	15.9	5.7	100.000
