# Supplementary figures and images for: Automated Synthesis and Initial Evaluation of (4′-Amino-5′,8′-difluoro-1′H-spiro[piperidine-4,2′-quinazolin]-1-yl)(4-[18F]fluorophenyl)methanone for PET/MR Imaging of Inducible Nitric Oxide Synthase
Source: Mol Imaging. 2021 Jul 8;2021:9996125. doi: 10.1155/2021/9996125 (PMC8328489; doi:10.1155/2021/9996125)

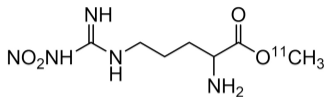

L-[<sup>11</sup>C]NAME

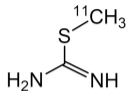

[<sup>11</sup>C]MITU

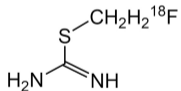

[<sup>18</sup>F]FEITU

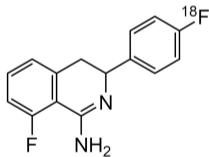

[<sup>18</sup>F]FFDI

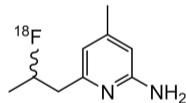

[<sup>18</sup>F]iNOS-9

Supplement: Supplementary Materials — See supplementary material for the structures of PET tracers in iNOS research mentioned in this article, the NMR spectra or LC/MS chromatogram of [18F]FBAT precursor or standard, and the retention time (tR) of [18F]FBAT in HPLC analysis. Suppl. Fig. 1: examples of PET tracers in iNOS research. Suppl. Fig. 2: (A) NMR spectra of [18F]FBAT precursor. (B) LC/MS chromatogram of [18F]FBAT precursor. Suppl. Fig. 3: (A) NMR spectra of FBAT standard. (B) LC/MS chromatogram of FBAT standard. Suppl. Fig. 4: (A) the retention time (tR) of [18F]FBAT in semipreparative HPLC was 10.07 min. (B) The retention time (tR) of [18F]FBAT in HPLC analysis was 14.38 min. (C) The retention time (tR) of authentic FBAT in HPLC analysis was 13.93 min. [file 9996125.f1.zip › Suppl_Fig_1.pdf]

**(A)**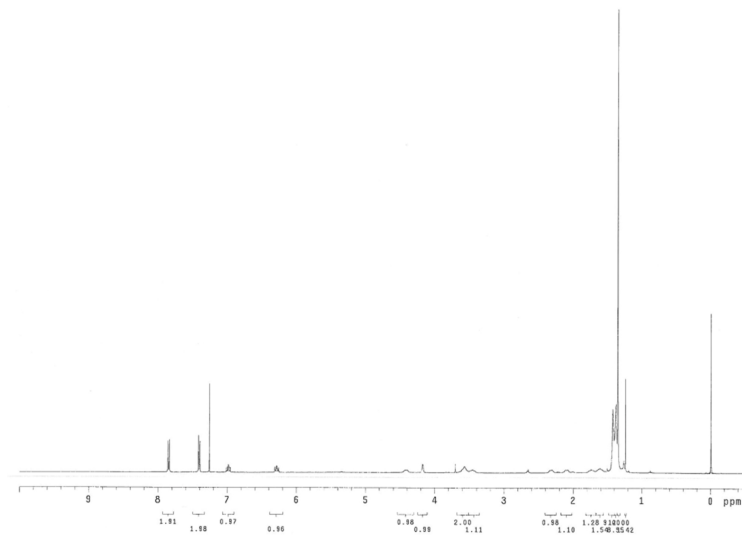**(B)**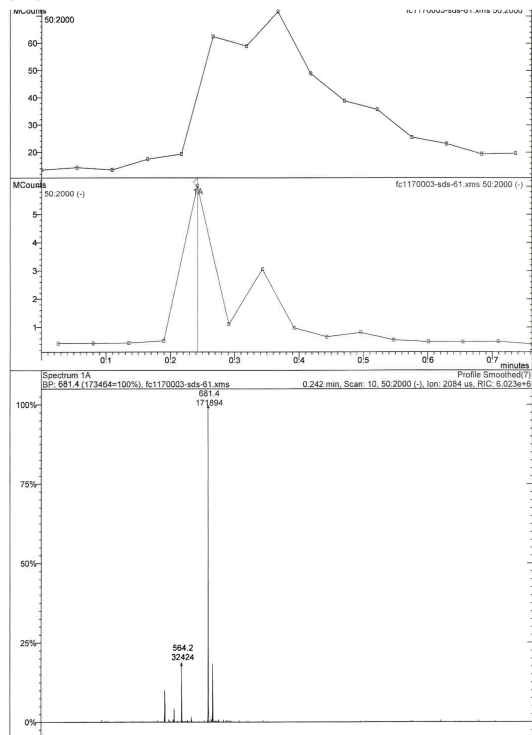

Supplement: Supplementary Materials — See supplementary material for the structures of PET tracers in iNOS research mentioned in this article, the NMR spectra or LC/MS chromatogram of [18F]FBAT precursor or standard, and the retention time (tR) of [18F]FBAT in HPLC analysis. Suppl. Fig. 1: examples of PET tracers in iNOS research. Suppl. Fig. 2: (A) NMR spectra of [18F]FBAT precursor. (B) LC/MS chromatogram of [18F]FBAT precursor. Suppl. Fig. 3: (A) NMR spectra of FBAT standard. (B) LC/MS chromatogram of FBAT standard. Suppl. Fig. 4: (A) the retention time (tR) of [18F]FBAT in semipreparative HPLC was 10.07 min. (B) The retention time (tR) of [18F]FBAT in HPLC analysis was 14.38 min. (C) The retention time (tR) of authentic FBAT in HPLC analysis was 13.93 min. [file 9996125.f1.zip › Suppl_Fig_2.pdf]

**(A)**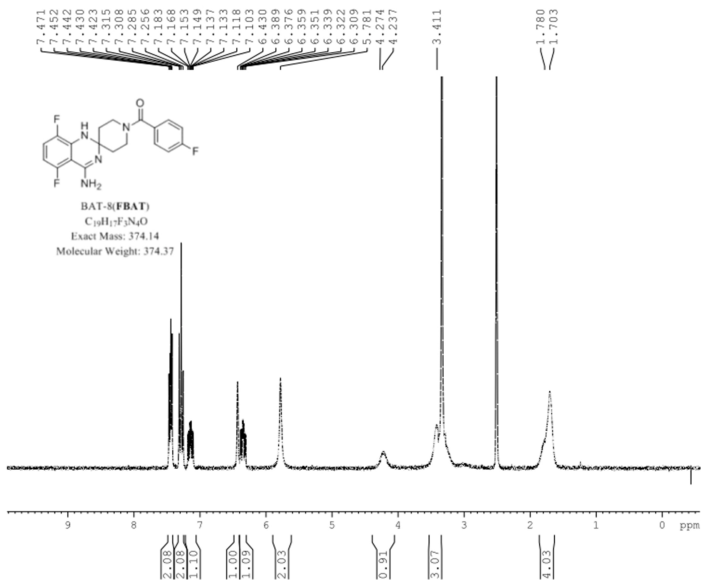**(B)**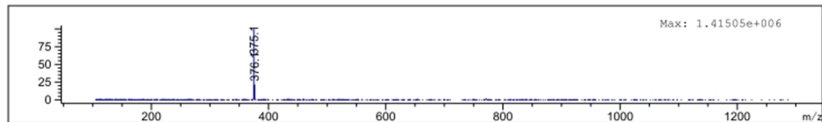

Supplement: Supplementary Materials — See supplementary material for the structures of PET tracers in iNOS research mentioned in this article, the NMR spectra or LC/MS chromatogram of [18F]FBAT precursor or standard, and the retention time (tR) of [18F]FBAT in HPLC analysis. Suppl. Fig. 1: examples of PET tracers in iNOS research. Suppl. Fig. 2: (A) NMR spectra of [18F]FBAT precursor. (B) LC/MS chromatogram of [18F]FBAT precursor. Suppl. Fig. 3: (A) NMR spectra of FBAT standard. (B) LC/MS chromatogram of FBAT standard. Suppl. Fig. 4: (A) the retention time (tR) of [18F]FBAT in semipreparative HPLC was 10.07 min. (B) The retention time (tR) of [18F]FBAT in HPLC analysis was 14.38 min. (C) The retention time (tR) of authentic FBAT in HPLC analysis was 13.93 min. [file 9996125.f1.zip › Suppl_Fig_3.pdf]
